# Performance-related feedback as a strategy to overcome spontaneous occupational stereotypes

**DOI:** 10.1177/17470218231196861

**Published:** 2023-09-08

**Authors:** Eimear Finnegan, Alan Garnham, Jane Oakhill

**Affiliations:** 1 School of Psychology, University of Sussex, Brighton, UK; 2 School of Psychological Sciences and Health, University of Strathclyde, Glasgow, UK

**Keywords:** Occupational stereotypes, performance feedback, stereotype reduction

## Abstract

This article investigates the use of performance-related feedback as a strategy for overcoming spontaneous occupational stereotyping when certain social role nouns and professional terms are read. Across two studies participants were presented with two terms: a role noun (e.g., surgeon) and a kinship term (e.g., mother) and asked to quickly decide whether both terms could refer to the same person. The feedback training involved telling participants whether their responses were correct or incorrect and providing them with their cumulative percentage correct score. In the absence of feedback, responding to stereotype-incongruent pairings was typically slower and less accurate than in stereotype-congruent and neutral conditions. However, the results demonstrated that performance significantly improved to stimuli on which participants received the feedback training (Experiment 1), and to a novel set of stimuli (Experiment 2). In addition, the effects were still evident 1 week later (Experiment 2). It is concluded that performance-related feedback is a valuable strategy for overcoming spontaneous activation of occupational stereotypes and can result in lower levels of stereotype use.

Despite widespread commitment to the pursuit of gender equality in many fields, including that of work, occupational stereotypes continue to influence expectations about who will enter which professions. The reason is that many occupations are stereotyped for which gender is more likely to enter them: Stereotyping an occupation by associating a typical gender with it is a much more restricted phenomenon than gender stereotyping itself, which works in the opposite direction by potentially associating a large number of characteristics, often detrimentally, with one gender or another. Note, however, that gender stereotypes (e.g., physical strength) may affect how genders are associated with occupations, so the two phenomena are, to some extent, interrelated. In English, a natural gender language in which gender is not always explicitly marked, these stereotypes can influence inferences based on a person’s occupation or on who is likely to enter a certain occupation. In cases where unequivocal gender information about a character is lacking, perhaps following the use of an unmarked social/occupational role noun (e.g., secretary, carpenter), prior knowledge in the form of an occupational stereotype may be employed to supply a default gender (Carreiras et al., 1996). For this reason, people come to expect, for example, surgeons to be male and nurses to be female.

Researchers have frequently used role nouns as part of a match–mismatch paradigm in which participants are presented with a role noun followed by either stereotype-consistent (*the surgeon . . . he*) or stereotype-inconsistent (*the surgeon . . . she*) information. Unfailingly, processing difficulty is apparent in the mismatch condition relative to the match condition—typically evidenced by slower judgement or reading times and an increased tendency, where a judgement is required, to reject mismatch pairings as incorrect, when they are in fact entirely valid (e.g., Carreiras et al., 1996; Garnham et al., 2002; Kreiner et al., 2008; Oakhill et al., 2005). Such findings are thought to reflect difficulty in integrating the unexpected gender information into the reader’s mental model (Sato et al., 2013). In this article, we investigate the deeply ingrained stereotypes associated with many social role nouns in English and propose the use of performance-related feedback as a strategy for overcoming such persistent, and often unwanted, stereotyping.

Stereotypes are well-learned sets of associations that can automatically activate when a member of a target group (or a description of such a person) is encountered (Devine, 1989). How stereotypes are learned is an interesting question, though any connection between how they are learned and how they might be overcome remains to be established. Wood and Eagly (2012) provide an insightful historic account of the development and maintenance of gender-related stereotypes, and Hinton (2017) provides a Bayesian account of how individuals learn current descriptive stereotype norms. However, this account does not address what Eagly calls the injunctive (and often insidious) aspect to these norms. The injunctive norms, and indeed incorrect information about descriptive norms, perhaps generated by applying historical information to the present day, might be part of the top–down information that produces the Bayesian priors in Otten et al.’s (2017) account of stereotyping.

In our case the target groups of the stereotypes we are addressing are occupations. Much research has established that this activation can occur without a perceiver’s intention or awareness (Bargh, 1997; Bargh et al., 1996; Devine, 1989; Fiske & Neuberg, 1990; Macrae et al., 1994, 1995; Winter & Uleman, 1984), but there is less consensus about the inevitability of such stereotype activation.

Some recent evidence (e.g., Falbén et al., 2019; Tsamadi et al., 2020) suggests that in a simple sequential priming task, where stereotype information is present in the second (target) stimulus, faster processing in cases where the stereotype information is congruent with gender information in the first (priming) stimulus arises from a response bias generated by the first stimulus, not via enhanced efficiency of processing of stereotype-consistent (vs stereotype-inconsistent) stimuli. Hence, there is no evidence, in these studies, that the stereotype is activated. However, these findings are not directly relevant to our study, because in our study the first stimulus presented is stereotyped, not the second, and Falbén and Tsamadi’s Hierarchical Drift Diffusion Modelling is based on the assumption that gender information in the first stimulus is activated.

While early research suggested that perception of group members (e.g., people in certain occupations) leads to automatic activation of stereotypic knowledge relevant to that group (Fiske, 1998), more recent work has identified factors that can moderate stereotype activation, for example, availability of cognitive resources (Gilbert & Hixon, 1991), and the strength and accessibility of the stereotype (Fazio, 1990, 1995; Fazio et al., 1995; Lepore & Brown, 1997; Wittenbrink et al., 1997).

Past efforts to reduce stereotyping have distinguished between overcoming initial stereotype activation and overcoming subsequent stereotype application. These two processes are closely tied to automatic and controlled processing; stereotype activation is thought to take place automatically and stem from increased cognitive accessibility of traits and features connected with a specific group, while stereotype application is typically under conscious control and involves the actual use of stereotypes in response to a group member (Kawakami et al., 2005). While strategies are typically aimed at reducing either stereotype activation or stereotype application, both types of processes are relevant to the current research in which we investigate a strategy for overcoming the initial, spontaneous activation of occupational gender biases so as to ultimately result in lower levels of stereotype application. This stereotype application was assessed using the judgement paradigm of Oakhill et al. (2005).

## Occupational stereotype reduction in language processing

Oakhill et al. (2005) investigated whether gender biases are evoked for single words (role names), and the extent to which such biases can be overcome. They devised a task in which participants were presented with two terms: a role name that was either definitionally male or female, or stereotype biased (e.g., *princess, beautician*, respectively) and a kinship term that was definitionally gendered (e.g., *father, mother*). The participants’ task was to quickly decide whether both terms could be used to refer to a single person. To perform well they needed to take definitional gender into account (e.g., that a mother is female), but suppress occupational stereotypes that are likely to be activated (e.g., that *most* beauticians are female, but not all of them). If participants are successful, the final judgement will not be affected by occupational stereotyping, but, if unsuccessful, stereotype application will be evident.

Oakhill et al. (2005) conducted six experiments using this judgement task, in which stimuli presentation and instruction details were varied. Across all experiments, performance was moderated by the stereotype bias of the role nouns, i.e., participants more frequently rejected word pairs in which the occupational gender bias of the role noun was incongruent with the gender of the kinship term (e.g., *electrician, mother*) as opposed to congruent (e.g., *electrician, father*). This effect was still evident (although to a lesser extent) when participants were provided in their instructions with a strategy aimed at helping them overcome stereotype biases. This strategy simply involved explicitly reminding them that nowadays jobs are not typically restricted to a particular gender and that they should carefully consider whether the occupation mentioned in the first term presented (i.e., the role noun) could be occupied by a man, a woman, or either (Experiment 4). These results provide strong evidence for an automatic component to responding as participants displayed difficulty in suppressing the gender bias associated with the presented role nouns, despite explicit instructions to do so. The authors posit that stereotypical information about gender associated with role nouns is typically incorporated into a mental representation of a person described by such a role name “immediately (and probably automatically)” (p. 982)—even when it is counter-productive to task performance.

Although our results were robust, indicating an effect of the processing of the first term on the second, it has more recently been suggested (e.g., Kidder et al., 2018; White et al., 2018 see also Falbén et al., 2019; Tsamadi et al., 2020) that speeded and/or more accurate processing of primed information in a sequential priming task may be restricted to, or at least be more reliably found in, stimulus–response (S-R) tasks rather than stimulus–stimulus (S-S) tasks. In S-S tasks there is a semantic relation, at least on critical trials, between the prime and the target. In S-R tasks there is, in addition, a semantic relation between the stimulus and the specification of the response. For example, with a gender stereotyped noun as prime, gender-classification on the target would be an S-R task, whereas lexical decision on the target would be S-S. However, these observations are not as applicable to our task as they might initially appear to be, for three reasons. First, the response for our task is not defined in relation to the “target” (second word) alone but in terms of a relation between the two words (whether they can refer to the same person). So the task is not a simple priming task. Second, it is not immediately clear that the S-S versus S-R distinction, which is defined in terms of a response to the second word, can be applied to our task, and third, as we have already mentioned, we have found clear match–mismatch effects using this task in the past.

The automaticity of invoking the gender-related component of occupational role stereotypes is also supported by sentence comprehension research. In two eye-tracking studies, Duffy and Keir (2004) first observed a processing cost in the integration of a reflexive pronoun with a role noun that had an incongruent gender stereotype, e.g., “The *secretary* treated *himself* to a large sundae after finishing work.” In efforts to overcome this stereotype effect, they found that by explicitly stating the gender of the person who is being referred to using a stereotyped role noun, the subsequent difficulty in integrating stereotype-incongruent information disappears, e.g., “the electrician was a cautious woman who taught *herself* a lot while fixing the problem.” In a similar vein, Kreiner et al. (2008) discovered that presenting participants with sentences in which the reflexive pronoun *preceded* the stereotyped term helped to overcome any processing cost associated with the integration of the bias information, e.g., “After reminding *himself / herself* about the letter, the *minister* immediately went to the meeting at the office.” However, such strategies that involve manipulating prior context or explicitly providing gender information do not necessarily show that stereotyping itself has been lessened—but may simply show that processing of definitional gender information (in the form of a noun such as “woman” or a gender-marked pronoun) overrides gender assumptions from occupational stereotypes. While such strategies are useful to be aware of, for example, in producing written material, a clear need remains to identify a strategy to overcome stereotyping of occupations for gender when occupational role names are used on their own, without the disambiguating effect of explicit gender information. Once a role noun is encountered (and a stereotypical gender is likely activated), can a perceiver quickly realise that such terms do not reliably indicate gender in the same way that definitionally gendered terms do?

Two past studies examined this question using the judgement task of Oakhill et al. (2005), but achieved limited success. These studies involved strengthening counter-stereotypical representations of occupations by presenting participants with pictures of people working in counter-stereotypical roles (Finnegan et al., 2015b), or by inducing social norm-related pressure by suggesting that occupational stereotypes were commonly rejected by previous participants (Finnegan et al., 2015a). Despite some success at reducing stereotyped responding to critical, stereotype-incongruent, trials (e.g., *electrician, mother*), ultimately stereotype use persisted as performance on these pairings remained consistently poorer than to stereotype-congruent pairings. The results of the above studies provide evidence that occupational stereotypes are automatically activated when certain role names are read and that these stereotypes can have an impact on text processing. They also show that such effects can be partly overridden, at least temporarily. To build on this past work, we were interested in devising a more effective strategy that could lead to improved performance and also to examine the generalisability and durability of its effects.

## Performance feedback

In creating our feedback strategy aimed at overcoming spontaneous stereotype activation so as to result in low levels of stereotype application, we looked to previous literature on stereotype reduction to inform our procedure.

Given that automatic stereotyping is a by-product of an enormously efficient categorisation system that allows us to rapidly simplify information-rich environments (Operario & Fiske, 2004), it is unlikely that such category activation will be easily overcome. However, if we take stereotypes as being well-learned sets of association (Devine, 1989), then it could be logically argued that a successful strategy for overcoming occupational stereotyping should initially involve alerting a perceiver to the fact that they are succumbing to these stereotypes, thus creating awareness that they are making unreliable associations. Once awareness of automatic stereotype activation has been created, perceivers can then consciously decide whether or not to endorse this activation.

This theorising is in line with the work of Devine and colleagues, who posit that people must be aware of biases and concerned about the consequences of their biases if they are to overcome them. Furthermore, they argue that “breaking the habit” of implicit bias requires people to learn about when bias may be activated and how to replace the biased responses with others that are in line with their non-prejudiced goals (Devine, 1989; Devine et al., 2012; Devine & Monteith, 1993; Devine et al., 1991; Monteith, 1993). In line with these ideas, Devine et al. (2012) and Lai et al. (2014, 2016) looked at various strategies aimed at both long-term and short-term reductions in implicit race bias, with both sets of researchers concluding that a multifaceted strategy was needed (see also, Paluck & Green, 2009). Overcoming implicit bias (commonly defined as unconscious bias that activates without one’s awareness or control) could logically be seen as akin to overcoming the spontaneous and automatic occupational gender bias that may come to mind when presented with a term such as “surgeon” in the paradigm we propose to use. We therefore posit that a multifaceted approach to implicit bias reduction should also prove effective in relation to spontaneous occupational stereotyping and note that Paluck and Green (2009) report that, for interventions aimed at individuals, strategies that achieved success involved “instruction, expert opinion and norm information, manipulating accountability, consciousness-raising, and targeting personal identity, self-worth, or emotion” (p. 347).

With the above in mind, we opted to investigate the use of performance-related feedback as a means of overcoming spontaneous occupational stereotyping. First, our strategy was to present participants with immediate and direct performance-related feedback after each judgement to indicate whether their response was *Correct* or *Incorrect* (remember that the task was to judge whether two terms could be applied to the same person). A primary aim of this feedback was to increase awareness of stereotyping by signalling their incorrect responses to each participant. It was anticipated that the feedback would alert participants to their personal occupational stereotyping tendencies, thus triggering control processes and helping to reduce subsequent levels of stereotype application. The feedback should essentially remind participants that certain roles can be fulfilled by either sex (irrespective of stereotype norms), assist them in learning from their mistakes, and lead to improved performance on subsequent trials. Such an approach would align with the successful “consciousness-raising” strategies identified by Paluck and Green (2009) and acknowledges the key role of awareness in overcoming bias that was highlighted by Devine and colleagues (see above).

Second, our strategy was to present participants with their cumulative percentage score after each judgement response. The aim of showing this score was to increase task engagement and motivation by allowing participants to easily gauge and track their performance as they progressed through the trials. If participants are motivated to avoid occupational stereotypes (or even just to be perceived as avoiding them), it is possible that this cumulative feedback may additionally induce behaviour change if they realise they are making stereotyped responses. These reactions would also be in line with previously successful strategies identified by Paluck and Green (2009), outlined above.

Finally, the proposed feedback strategy sought to directly tackle stereotypes as “overlearned associations” by incorporating extensive practice into the training. By giving feedback on a relatively large number of trials (152) it was hypothesised that ongoing practice would help participants break the stereotyping habit by repeatedly suppressing stereotype activation and activating counter-stereotype associations so they could make a correct response. Such repetition should encourage the formation of new representations (Kawakami et al., 2000). Indeed, Shiffrin and Schneider (1977) argued that with considerable and consistent training, automatic responding to a particular stimulus could be “unlearned” and newer responses could take their place.

Overall, the stereotype reduction strategy was intended to both create awareness of occupational stereotype biases and to facilitate overcoming these biases, as participants should quickly realise when they are responding in a stereotyped manner and work out how to avoid doing so (by accepting that males and females can actually do many jobs that are stereotypically associated with one sex). In line with previous strategies that successfully leveraged multiple mechanisms to achieve stereotype reduction, we posited that several components (primarily awareness, motivation, and practice) would likely be working together to result in stereotype reduction.

In relation to the question of the mechanism by which feedback has its effect, ideas from the reinforcement learning literature (e.g., Frank et al., 2007; Lindström et al., 2015) could potentially help to provide a more detailed account of how learning occurs in feedback-based tasks. We do not explore these issues further here as the “how” of learning is not one of the questions that we are directly addressing.

## Hypotheses

As a reminder, the participants’ task in this study was to judge as quickly as possible whether two terms could be used to refer to one and the same person. In order to respond successfully, participants were required to suppress occupational stereotypical gender information but to take account of definitional gender information at least on some trials. Participants responded to three blocks of such trials, with performance-related feedback offered only in the second block (in the Training condition). The aim of Experiment 1 was to further investigate whether stereotyped information associating gender with occupations is automatically elicited from single words (as has been found in previous work, e.g., Banaji & Hardin, 1996; Oakhill et al., 2005), and to examine whether this stereotyping can be attenuated by the introduction of performance-related feedback. If successful, a follow-up study would be conducted to explore whether the effects of training would extend to novel stimuli and whether reduced levels of stereotyping would also be evident 1 week later.

It was hypothesised, in keeping with previous findings (Garnham et al., 2002; Irmen, 2007; Kreiner et al., 2008; Oakhill et al., 2005; Reynolds et al., 2006), that participants would initially respond more slowly and less accurately to stereotype-incongruent word pairs (e.g., *nurse/father*) than to stereotype-congruent word pairs (*nurse/mother*). However, with the introduction of feedback, the processing cost associated with the stereotype-incongruent pairs should be attenuated (as evidenced by higher accuracy and faster reaction times to these trials), and that this improved performance would also extend into Block 3 (once feedback had been discontinued).

## Experiment 1

### Method

#### Participants

A total of 81 monolingual, native English speakers (38 male, 43 female) from the student population of the University of Sussex took part.^1^ They were assigned to one of two conditions (51 in the Training condition and 30 in the No Training condition). Participants’ ages ranged from 18 to 31 years (*M*: 20.56; *SD*: 2.76) and they received either £5 or course credits for taking part in the session, which lasted approximately 45 min. Ethical approval for both experiments in this article was obtained from the University of Sussex, Life Sciences and Psychology Cluster-based Research Ethics Committee under reference JOEF0311. The Committee follows the British Psychological Society guidelines for ethics on human participant testing, which are in line with the Helsinki Declaration. All participants signed a consent form prior to participating.

### Materials

#### Gender-biased occupational role nouns

Role nouns were identical to those described in Finnegan et al. (2015a) and consisted of the 12 most highly male-biased, the 12 most highly female-biased, and the 12 most closely neutrally rated role nouns from norms compiled by Gabriel et al. (2008). A full list of the stereotyped terms used, and their associated bias ratings is provided in the online Supplementary Material 1A. As explained in Finnegan et al. (2015a), the bias ratings of the male items have a narrower range than those of the female items (11.10% from strongest [*M* = 88.24%] to weakest [*M* = 77.14%] vs 17.55% from strongest [*M* = 13.27%] to weakest [*M* = 29.22%], respectively, *t*(22) = 3.53, *p* = .002), thus indicating that the male-biased terms were judged to be more strongly stereotype biased than the female-biased items. Despite this difference, we used this set of terms as it was deemed more pertinent to include the more strongly biased role nouns for each sex than to choose two sets with matching typicality (as it was hypothesised that evidence of overcoming stereotyping to the strongest exemplars should logically extend to weaker exemplars). Ratings of the neutral terms had a very narrow range of 5.29% around the 50% neutral mark (*M* = 52.94% to *M* = 47.65%).

#### Kinship terms

As in past studies (Finnegan et al., 2015a, 2015b; Oakhill et al., 2005), six kinship terms (*father, mother, brother, sister, uncle, aunt*) were used as one of the terms in the word pairs. Importantly, these words incorporate a specific gender into their definitions, e.g., the term *mother* refers to females.

#### Critical word pairs

By combining the 12 male-biased, 12 female-biased, and 12 neutral role nouns with the 6 kinship terms, a set of occupational stereotype-congruent (e.g., *pilot/brother, nurse/sister*), stereotype-incongruent (e.g., *pilot/sister, nurse/brother*) and neutral pairings (e.g., *artist/father, artist/mother*) was produced.^2^ Each of the 12 male-biased, 12 female-biased, and 12 neutral role terms was combined once with each of the 6 kinship terms resulting in 72 word pairs in each congruency condition, totalling 216 critical trials. Thus, there were the same number of congruent and incongruent word pairs, though in the real-world, female engineers are less common than male engineers and that is one factor that might affect participants’ initial responding in the experiments. However, correct responses to critical trials were not probabilistic. There was always a correct or incorrect response. For example, responding *yes* that a pilot and sister can be the same person is correct, but responding *no* that they cannot be the same person is incorrect.

#### Filler trials

These trials were also constructed as outlined in Finnegan et al. (2015a), i.e., 240 word pairs were created by pairing the 6 kinship terms with role nouns that are also gender-specific by definition (e.g., *waitress, waiter*) so as to create gender unambiguous pairings to which participants could respond *yes* or *no* with relative ease and certainty. These role nouns were sourced from Hamilton (2008) and Kennison and Trofe (2003). A complete list of the filler terms is provided in the online Supplementary Material 1B.

#### Item overview

Participants were presented with 456 word pairs, divided into three equal blocks of 152 trials. The stereotyped terms appeared twice in each block, once with a male kinship term and once with a female kinship term, i.e., in both the congruent and incongruent conditions. The six kinship terms appeared with the critical items an equal number of times in each block. In total, 276 items, including all critical items, were intended to elicit a *yes* response, while 180 required a *no* response.

The set of items used, including the balance of *yes* and *no* items, is based on two previously published studies (Finnegan et al., 2015a, 2015b). This parity allows an informed comparison of the results of the different studies. In relation to the imbalance in correct responses, and whether this imbalance might produce a response bias to *yes* responses, we expected a substantial number of *yes* items to receive *no* responses (the counter-stereotype items), particularly prior to training, but we did not expect many *no* items to receive *yes* responses. Thus, we expected a closer balance of *yes* to *no* responses than the *yes/no* ratio for correct responses would suggest. There would be no reason for participants to believe they were being encouraged to answer *yes* rather than *no*, and hence no reason to think that any aspect of our results might be explained in terms of response bias.

#### Performance-related feedback

Performance-related feedback was presented to participants as a strategy aimed at reducing levels of occupational stereotype application. This feedback was provided after each response in Block 2 of the judgement task, in the Training condition only. It consisted of a statement of accuracy in which participants were informed whether their response was *Correct* or *Incorrect*, along with a report of their cumulative percentage correct score. Therefore, feedback consisted of a statement such as “Correct! 75% average correct.” It is important to note that this feedback was not simply stereotype disconfirming, but indicated the correct answer to the question: can these two terms refer to the same person? Participants in the No Training condition did not receive feedback in Block 2.

### Design

Role terms were presented one at a time in the centre of a computer screen for 1,000 ms, and then immediately replaced by a kinship term (interstimulus interval of 0). The kinship term remained on the screen until a response was made.^3^ In the Training condition, after a response had been made feedback immediately appeared on-screen (0 delay) and remained for 1,000 ms. In both conditions there was a 500-ms delay before the onset of the next trial. Three fixed sets of word pairs were created to form the blocks of the experiment, and the order in which these blocks were presented to participants was counter-balanced. In each block, trial order was randomised separately for each participant using the standard E-Prime procedure. Participants made a judgement about each word pair using a button box with one button clearly marked *Y* for *yes* and another *N* for *no*. The proportion of correct answers and response times (RTs) for judgements to correct trials were analysed.

### Procedure

Participants were tested individually in a quiet laboratory with on-screen instructions asking them to decide (without excessive deliberation) whether the two terms presented could refer to the same individual. Two examples of word pairs were provided—one that required a *yes* response and one that required a *no* response. In the Training condition participants were further informed that they would receive feedback in the second block of judgement trials, and it was explained to them what this feedback entailed. All instructions and examples were next repeated verbally. Finally, in both conditions, a short practice session was held (using a representative sample of fillers and critical word pairs) to familiarise the participants with the experimental task. Eight trials were presented, using role terms that were not subsequently used in the experimental blocks.

### Results

#### Data screening

Prior to the analyses, data for word pairs that contained the neutral term *adolescent* were excluded as accuracy of responses to such pairs was low, resulting in only 66% correct responses in Block 1 compared with >86% accuracy for all other neutral role nouns. As in Finnegan et al. (2015a, 2015b), it is posited that this finding may be explained by age considerations as opposed to occupational stereotyping—as the term *adolescent* was paired with kinship terms that generally imply an older generation, e.g., *uncle, aunt, mother, father*, participants showed more difficulty with pairings containing these latter terms, e.g., *adolescent/father* than those without them, e.g., *adolescent/brother*, despite both being possible combinations. This exclusion resulted in the removal of 1.32% of the data.

#### Analysis

Accuracy of judgements and RTs for correct trials only were analysed using two mixed analysis of variances (ANOVA), one with participants treated as the random variable and one with items treated as the random variable. In the by-participants analysis (*F*_1_), the mixed ANOVA had three repeated factors—stereotype bias of the role name (Occupational Stereotype: Male/Female/Neutral), gender of the kinship term (Kinship term gender: Male/Female), and block of trials (Block: Block 1/Block 2/Block 3). Participant sex^4^ (Male/Female) and Training condition (Training/No Training) were included as between-subject factors. In the by-items analyses (*F*_2_), (Occupational) Stereotype was included as a between-items factor, while Kinship term gender, Block, Participant Sex, and Training condition were included as within-item variables. In all analyses, where sphericity was not satisfied, Greenhouse–Geisser (when ε < 0.75) or Huynh–Feldt (ε > 0.75) corrected degrees of freedom and *p* values are presented (as recommended by Girden, 1992). With the paired *t*-tests, within-subject or within-item effect sizes were estimated using Cohen’s *dz* while with the independent-samples *t*-tests, estimates of between-subject or between-item effect sizes were estimated using Cohen’s *d*. For ANOVAs, partial eta-squared (η_p_^2^) is reported.

#### Congruency

Note that an interaction of Stereotype by Kinship term gender is essentially an effect of Congruency as it is the combination of the levels of these two factors that result in the three critical conditions—congruent, incongruent, and neutral. Therefore, all Stereotype by Kinship term gender interactions are referred to as effects of Congruency (though principally in relation to the male and female stereotyped terms).

### Experiment 1: accuracy

The main questions of interest were (1) whether performance to incongruent word pairs was significantly lower in Block 1 than to congruent or neutral pairings and, if so, (2) whether performance to these incongruent pairings improved across blocks when performance-related feedback was provided in the Training condition.

A main effect of Training condition was observed, *F*_1_(1, 77) = 6.89, *p* = .010, η_p_^2^ = .08; *F*_2_(1, 32) = 159.36, *p* < .001, η_p_^2^ = .83, with participants achieving higher accuracy averaged over all three blocks when the feedback training was provided (*M* = 95.6%) than when it was not (*M* = 90. 1%). However, of more interest was the significant three-way interaction of Congruency by Block by Training condition, *F*_1_(1.88, 144.49) = 4.92, *p* = .010, η_p_^2^ = .06; *F*_2_(4, 64) = 11.59, *p* < .001, η_p_^2^ = .42.

**Figure 1.**
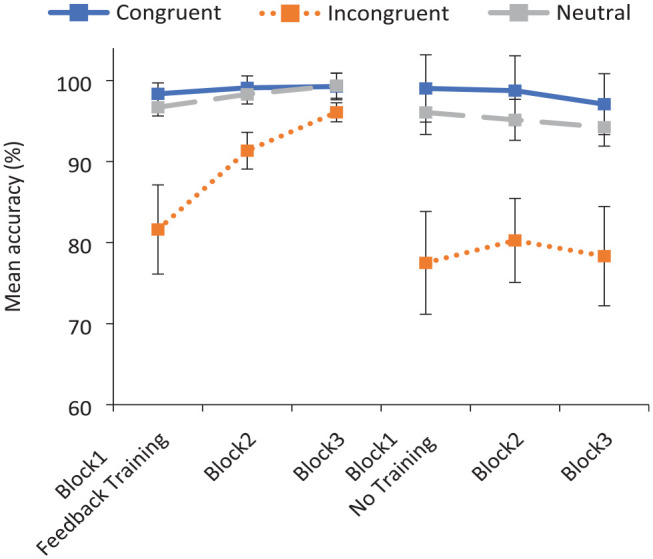
Experiment 1: mean accuracy to critical word pairs across blocks in the training and no training conditions. Error bars indicate the 95% confidence intervals.

The Congruency by Block by Training condition interaction was primarily driven by variable performance on stereotype-incongruent trials, with ceiling effects evident in response to stereotype-congruent and neutral pairings across conditions (see Figure 1). When training was provided, accuracy to incongruent pairings was found to rise steadily across blocks, stemming from the provision of feedback in Block 2. However, accuracy to the incongruent word pairs did not improve in the Control condition when no feedback was provided.

To establish whether Block 1 (pre-training) performance to incongruent word pairs was significantly lower than that of congruent or neutral pairings, one-tailed *t*-tests were conducted (paired samples by-participants, and independent samples by-items) across conditions. It was found that performance was significantly lower to stereotype-incongruent than to stereotype-congruent pairings, *t*_1_ (80) = 6.43, *p* < .001, *dz* = .72; *t*_2_ (52.68) = 13.98, *p* < .001, *d* = 2.85, and neutrally rated pairings, *t*_1_ (80) = 6.58, *p* < .001, *dz* = .73; *t*_2_ (63.26) = 11.65, *p* < .001, *d* = 2.36.

Next, to examine whether performance on the stereotype-incongruent pairings improved when feedback training was provided, a second set of ANOVAs was conducted on the incongruent conditions alone, using a Training condition (Training vs No Training) by Block (1 vs 3) design. A significant interaction of Training condition by Block was revealed, *F*1 (1, 79) = 10.98, *p* < .001, η_p_^2^ = .12; *F*2 (1, 23) = 65.03, *p* < .001, η_p_^2^ = .74. While there was no reliable Block 1 difference between the Training and No Training conditions, the Block 3 difference was highly significant in both sets of analyses, reflecting the substantial improvement across blocks when feedback training was provided, relative to the control condition, *t*_1_ (31.27) = 3.20, *p* = .001, *d* = 1.14; *t*_2_ (23) = 11.10, *p* < .001, *dz* = 2.27.^5^

Finally, to investigate the magnitude of Block 1–Block 3 improvement to incongruent pairings in the Training condition, a final set of *t*-tests was conducted, *t*_1_ (50) = 4.82 *p* < .001, *dz* = .67; *t*_2_ (23) = 10.97 *p* < .001, *dz* = 2.24. These results will be discussed further in the “General discussion” section when comparing the efficacy of this stereotype reduction strategy with previous strategies.

### Experiment 1: RTs

RTs for all incorrect responses were identified and excluded (representing 7.50% of the total data) as were extreme RTs: those below 150 ms or above 4,000 ms (representing a further 2.11%). In total, therefore, 9.61% of the data was excluded. Next, the Participant by Block mean was calculated for each participant. Data points of 2.5 *SD*s above or below the Participant by Block means were replaced with the relevant upper or lower cut-off point.

**Figure 2.**
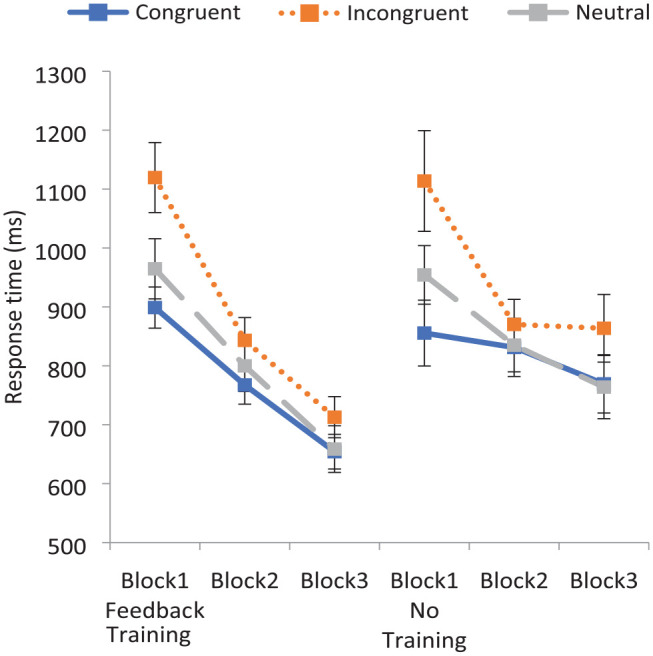
Experiment 1: mean response times to critical word pairs across blocks in the training and no training conditions. Error bars indicate the 95% confidence intervals.

Unlike in the accuracy data, a main effect of Training condition was observed in the by-items analysis only,^6^ *F*_1_ (1, 77) = .849, *p* *=* .360; *F*_2_ (1, 32) = 31.59, *p* < .001, η_p_^2^ = .50, with faster RTs when training was provided (816 ms) than when it was not (868 ms). A two-way interaction of Congruency by Block was also found, *F*_1_ (4, 308) = 13.56, *p* < .001, η_p_^2^ = .15; *F*_2_ (4, 64) = 6.73, *p* < .001, η_p_^2^ = .30. However, contrary to expectations, this interaction was not different across training conditions, as a significant three-way interaction of Congruency by Block by Condition failed to emerge, *F*_1_(4, 308) = .947, *p* *=* .437; *F*_2_(4, 64) = .517, *p* = .724. To examine the pattern of responding across conditions more closely, this information is displayed in Figure 2.

It is apparent that RTs improved across blocks to incongruent pairings (and indeed to all pairings) even when feedback training was not provided. In this case, the improvement in RTs across blocks is likely due to practice effects with participants speeding up as they got used to the task (as no corresponding increase in accuracy was found).

As with the accuracy data, *t*-tests revealed that Block 1 RTs to incongruent word pairs were significantly slower than to congruent, *t*_1_ (80) = 8.57, *p* < .001, *dz* = .95; *t*_2_ (76.47) = 8.49, *p* < .001, *d* = 1.73, or neutral pairings, *t*_1_ (80) = 5.94, *p* *<* .001, *dz* = .66; *t*_2_ (88.53) = 4.35, *p* < .001, *d* = .90, across conditions.

A final set of ANOVAs were conducted on the incongruent data alone so as to more closely examine performance on these pairings, using a Training condition (Training vs No Training) by Block (1 vs 3) design. A significant Training condition by Block interaction was revealed, *F*_1_ (1, 79) = 5.77, *p* = .019, η_p_^2^ = .07; *F*_2_ (1, 23) = 10.82 *p* = .003, η_p_^2^ = .32. While no significant difference in Block 1 RTs emerged, *t*_1_ (79) = 0.30, *p* = .768; *t*_2_ (23) = 0.98, *p* = .337, the RT difference was significant in Block 3, *t*_1_ (79) = 2.22, *p* = .015, *d* = .50; *t*_2_ (23) = 3.72, *p* < .001, *dz* = .76, illustrating that RTs improved to a greater extent following the feedback training relative to the control condition.

While there was no evidence of a 3-way interaction in the overall data, this is likely due to the similar performance to stereotype-congruent and neutral pairings as RTs decreased steadily across all three blocks in both the Training and No Training conditions. In contrast, while RTs to stereotype-incongruent pairings greatly decreased in Block 3 after the feedback training, RTs did not decrease further when feedback was not supplied.

Finally, *t*-tests were conducted to investigate the magnitude of the Block 1–Block 3 improvement to incongruent pairings in the Training condition, *t*_1_ (50) = 10.53 *p* < .001, *dz* = 1.47; *t*_2_ (23) = 10.68, *p* < .001, *dz* = 2.18. These results will be discussed further in the “General discussion” section when comparing the efficacy of this stereotype reduction strategy with previous strategies.

#### Fillers

As responding to the filler trials was not the main focus of this work, only a descriptive analysis of results is presented in the main text. Details of the analyses can be found in the online Supplementary Material 2. An average of 95.49% accuracy was found across conditions in response to the definitionally matching word pairs (e.g., *prince/father*), but this fell to 86.08% with the definitionally mismatching pairings (e.g., *prince/mother*). However, as in Finnegan et al. (2015a, 2015b), this deterioration in accuracy on the mismatching pairs was driven by poorer accuracy on those involving definitionally male (78.57%) as opposed to definitionally female role names (93.60%). It is again posited that participants were interpreting certain male terms (e.g., *host, hero*) as generically applicable to both sexes until they were alerted to the fact that they should be stricter in their linguistic definitions—signalled through performance-related feedback in the Training condition or by encountering the definitionally female counterpart to a male term that may previously have been presented (e.g., once the term *heroine* has appeared, the term *hero* is less likely to be interpreted generically). Indeed, when performance on the male mismatching fillers was analysed in Block 3 following the feedback training, accuracy was much more in line with past findings (*M* = 90.46%) than when training was not provided (*M* = 76.0%). Although this finding was somewhat unexpected, it provides further support for the use of performance-related feedback as a means of alerting participants to their use of gender-related information.

The RT data tell a similar story. Average (correct only) reaction times to definitionally matching word pairs were faster (*M* = 915 ms) than to definitionally mismatching word pairs (*M* = 989 ms), with responses to the female mismatch pairings faster (*M* = 944 ms) than to the male mismatch pairs (*M* = 1,033 ms). This finding is again thought to be indicative of participants’ deliberation over certain definitionally male terms, which have female-specific counterparts, and which should, therefore, be taken as male specific, rather than generic. Again, participants in the Training condition were faster to respond to male definitionally mismatching pairings in Block 3 following the feedback training (*M* = 867 ms) than those in the control condition (*M* = 956 ms).

### Discussion of Experiment 1

Experiment 1 first sought to replicate the finding that occupational stereotype information is automatically evoked from single words (Banaji & Hardin, 1996; Oakhill et al., 2005). This was indeed found to be the case, as evidenced by significantly lower accuracy and slower RTs to stereotype-incongruent word pairs in Block 1 compared with stereotype-congruent and neutral word pairs.

The study next investigated whether such occupational gender biases can be overcome with the introduction of performance-related feedback. It was found that accuracy of judgements to stereotype-incongruent word pairs increased significantly across blocks in the Training condition but did not rise in the absence of this training. Similarly, RTs to stereotype-incongruent word pairs decreased significantly across blocks in the Training condition but, unexpectedly, also improved in the No Training condition. This latter finding suggests that participants may have benefitted from a general practice effect, speeding up in the judgement task as the experiment progressed. Nevertheless, RTs to stereotype-incongruent pairings in Block 3 of the study were ultimately faster in the Training condition than the No Training condition. As such, both the accuracy and RT data affirm the use of performance-related feedback as an effective means of overcoming spontaneous gender inferences so as to result in lower levels of occupational-stereotyped responding.

Overall, creating awareness of personal stereotyping tendencies through providing feedback on behaviour appears to be a straightforward means of overcoming the immediate activation of occupational stereotypes, and allows for reduced stereotype application, in the short term at least. While explicit training strategies have been used to tackle stereotype application in the past, this behavioural study is unusual in that a participant’s own performance accuracy was used as a means of both reminding and re-educating them about the social roles occupied by women and men. Although the efficacy of this stereotype-reduction strategy in the short term has been established, two important issues remained unaddressed. The first is whether the success of this training can be generalised beyond the stimuli on which feedback was received to other terms with an equally strong occupational-stereotype bias. The second is the durability of the training results—would they persist in the longer term? Experiment 2 examined these issues.

## Experiment 2: long term and transfer effects

Given the wide range of strategies aimed at overcoming automatic gender biases Lenton et al. (2009) conducted a meta-analysis to assess the relative success of different types of training. Although they report that automatic stereotypes are malleable and susceptible to certain single-session interventions, small effect sizes indicated that interventions do not guarantee success. However, given the over-reliance on single-session studies in the literature, they warn that their analysis essentially investigates the effectiveness of interventions at changing current output patterns, but not necessarily the underlying stereotypes. Equally, in their meta-analysis of prejudice reduction strategies, Paluck and Green (2009) highlighted an over-reliance on single-session studies aimed at “quick fixes” in past research.

The importance of more thorough appraisal of bias reduction strategies has been evidenced in recent work by Lai and colleagues (2014). They evaluated and compared 17 different interventions aimed at reducing implicit racial prejudice, with nine found to be successful. However, in a follow-up study, Lai et al. (2016) report that none of these previously successful interventions was effective following a delay ranging from several hours to several days. This research has important implications for the bias reduction literature as the authors concluded that short-term malleability in implicit preferences does not necessarily lead to long-term change, raising new questions about the flexibility and stability of implicit bias.

While value can certainly be gained from single-session studies in terms of identifying mechanisms behind change or highlighting specific scenarios when bias can be overcome, ideally strategies should be tested further to examine the parameters of their effects. Although the research of Lai and colleagues focused on implicit race-related preferences, their research is relevant across all prejudice and stereotype reduction literature given the lack of methodological rigour with which strategies are frequently assessed.

Few past studies have provided evidence that the effects of training extend beyond the immediate context and influence responses to novel stimuli. Such an investigation would allow researchers to assess whether effects are restricted to items that received the training/intervention, or importantly whether the training can more usefully be extended to related stimuli. The insufficient evaluation of past strategies is somewhat surprising given the implications that stereotyping at a young age may have for later life, e.g., in terms of career preferences (see Gottfredson’s, 1981, 2005 theory on career development). While exceptions to the above limitations exist (e.g., Blair et al., 2001; Dasgupta & Asgari, 2004, Study 2; Kawakami et al., 2000, Studies 2 and 3), with these points in mind, the current study aimed to provide a more comprehensive investigation of performance-related feedback as a stereotype reduction strategy by investigating the durability and generalisability of effects.

To investigate these issues, the experimental design of Experiment 2 was identical to that of Experiment 1, with three exceptions: (1) no control condition was included, so only a training condition was examined; (2) an entirely new set of male-biased, female-biased, and neutrally rated role terms was introduced in Block 3 so as to investigate whether the feedback training extended to a new set of stimuli; and (3) participants were asked to return to the laboratory 1 week after Session 1 to complete one final block of judgement trials, to assess the durability of the training effects. It was again hypothesised that participants would respond more slowly and less accurately to trials made up of stereotype-incongruent word pairs (e.g., *nurse/father*) than to stereotype-congruent word pairs (*nurse/mother*) in Block 1. However, on receipt of feedback in Block 2, it was hypothesised that this stereotyping effect would be reduced, and improved performance found in Block 3, despite the introduction of new stimuli. Finally, it was hypothesised that this improved performance would also be evident 1 week later with participants having learned to overcome initial activation of occupational gender stereotypes so as to lower levels of stereotype application.

### Method

#### Participants

In all, 36 monolingual, native English speakers (18 male, 18 female) from the student population of the University of Sussex participated. Participants’ ages ranged from 18 to 40 years (*M*: 19.89; *SD*: 4.53). They received either £8 or course credits for taking part in this two-part study. Session 1 lasted approximately 45 min, while Session 2 lasted approximately 15 min.

### Materials and design

#### Session 1

Session 1 consisted of three blocks of judgement trials, with performance-related feedback provided in Block 2 only. While the materials and design were largely as described in Experiment 1, the experimental stimuli were adapted to incorporate a new set of critical role nouns in Block 3. To achieve this end, an additional group of 12 male-biased, 12 female-biased, and 12 neutrally rated role terms was added to those used in Experiment 1. The majority of these terms were sourced from Gabriel et al. (2008), while some were selected from Kennison and Trofe (2003) (a full list of the original and new terms is provided in the online Supplementary Material 1A along with the mean bias ratings for each term). As role nouns with the strongest stereotype biases were originally selected for use in Experiment 1, the stereotype ratings of this new set were necessarily weaker (although the items were again chosen based on their relatively high stereotype ratings). Therefore, to create two groups of nouns with equal stereotype bias, the two sets of role nouns were combined and then individually matched based on their stereotype ratings. For example, the two strongest male stereotype role names were paired, followed by the next two strongest, and so on. One term from each pairing was presented in Blocks 1 and 2, while the other was presented in Blocks 3 and 4 (counter-balanced across participants). Finally, in this study the neutral term *swimmer* replaced *adolescent* as there was evidence that responses to the latter term were based on age considerations rather than gender considerations in Experiment 1.

#### Session 2

This session took place 1 week after each participant’s first session and involved one block of the judgement trials (one participant returned 8 days after Session 1, while all others returned 7 days after Session 1). This block was the same block of trials that participants had completed in Block 3 of Session 1, i.e., it contained the terms on which they had not previously received feedback. Items were again presented in a random order.

### Procedure

The experimental procedure was exactly as described in Experiment 1, but with instructions updated so as to remind participants to return 1 week later and take part in Session 2.

### Results

Data trimming followed by two mixed-design ANOVAs were conducted as outlined in Experiment 1, but with the Training factor removed and the Block factor updated so as to incorporate 4 blocks as opposed to 3. Although two different sets of critical role nouns were used for Blocks 1 and 2 versus Blocks 3 and 4, Block remained a within-items (or, strictly, a related-groups) factor in the following analyses, as all role nouns were individually matched for strength of stereotype bias, i.e., a related design was used. As previously, degrees of freedom were adjusted when sphericity was violated.

### Experiment 2: accuracy

**Figure 3.**
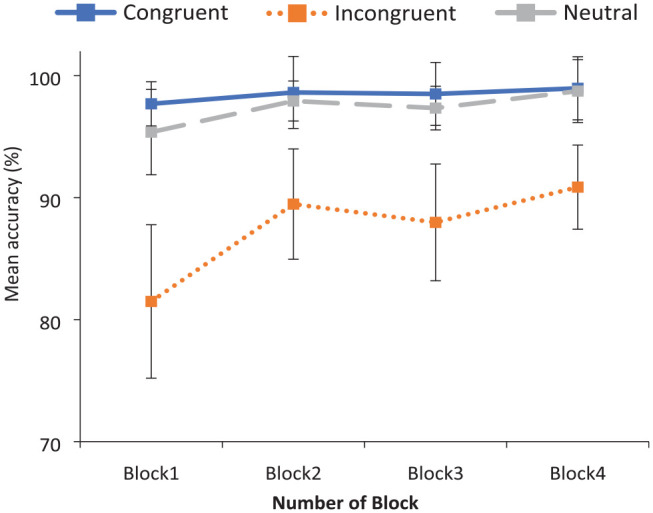
Experiment 2: mean accuracy to critical word pairs across blocks. Error bars indicate the 95% confidence intervals.

A main effect of Congruency was observed, *F*_1_ (1.02, 34.52) = 12.17, *p* = .001, η_p_^2^ = .26; *F*_2_ (2, 33) = 82.21, *p* < .001, η_p_^2^ = .83, with higher accuracy to stereotype-congruent (*M* = 98.45%) and neutral word pairs (*M* = 97.30%) than stereotype-incongruent word pairs (*M* = 87.45%). This difference in performance across congruency conditions was first evident in Block 1, reaffirming that spontaneous gender biases are elicited from certain social role nouns, stereotype-congruent vs stereotype-incongruent trials: *t*_1_ (35) = 4.13, *p* < .001, *dz* = .69; *t*_2_ (23) = 8.33, *p* < .001, *dz* = 1.70, and neutral versus incongruent trials: *t*_1_ (35) = 3.62, *p* < .001, *dz* = .60; *t*_2_ (23) = 7.91, *p* < .001, *dz* = 1.62. Importantly, there was also a significant interaction of Congruency by Block, *F*_1_ (3.33, 113.28) = 4.70, *p* = .003, η_p_^2^ = .12; *F*_2_ (4.02, 66.39) = 5.95, *p* < .001, η_p_^2^ = .27. Accuracy performance across blocks in each of the three congruency conditions is displayed in Figure 3.

#### Generalisability

To investigate whether the effects of the feedback training generalised to a novel set of items, accuracy in responding to stereotype-incongruent pairings in Block 1 versus Block 3 was first examined (i.e., pre-training accuracy vs post-training accuracy). A significant 6.5% increase in accuracy was found across these blocks, *t*_1_ (35) = 2.37, *p* = .024, *dz* *=* .39; *t*_2_ (23) = 4.41, *p* < .001, *dz* *=* .90, suggesting that performance-related feedback does successfully help reduce stereotype application to a new set of items.

#### Durability

To investigate the durability of this stereotype-reduction effect, performance accuracy in Blocks 3 and 4 (which was administered 1 week later) of the judgement trials was analysed. Accuracy was not found to deteriorate after 1 week, instead it rose a further 2.89% from Block 3 to Block 4, and this change was significant in the by-items analysis, *t*_1_ (35) = 1.57, *p* = .125, *dz* *=* *.26; t*_2_ (23) = 4.02, *p* = .001, *dz* = .82. This is convincing evidence for the durability of the feedback training. Accuracy was maintained 1 week after the initial training, and thus remained significantly greater than the pre-training levels of Block 1.

### Experiment 2: RTs

RTs below 150 ms, or above 4,000 ms were identified and removed before analysis (representing 2.61% of the total data in Session 1 and 2.76% in Session 2) along with times for incorrect responses (representing a further 7.52% in Session 1, 4.42% in Session 2). In total, therefore, 10.12% of the data in Session 1 and 7.18% in Session 2 were lost. Data points of 2.5 *SD*s above or below the Participant by Block means were replaced with the relevant upper or lower cut-off point.

**Figure 4.**
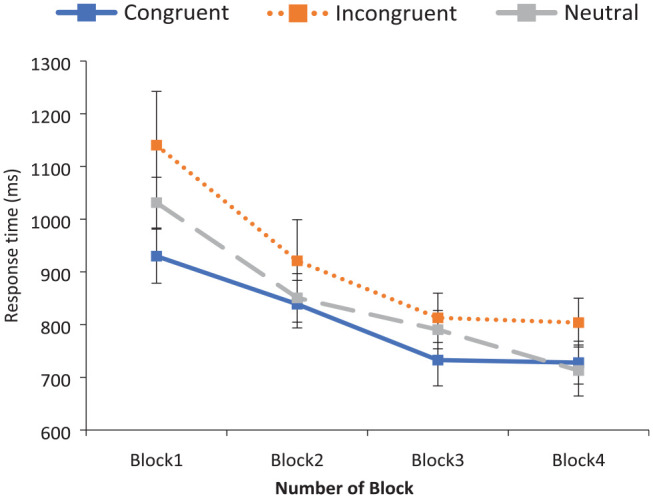
Experiment 2: mean response times to critical word pairs across blocks. Error bars indicate the 95% confidence intervals.

As with the accuracy data, there was a main effect of Congruency, *F*_1_ (1.42, 48.25) = 20.12, *p* < .001; *F*_2_ (2, 33) = 20.82, *p* < .001, η_p_^2^ = .56, driven by faster responses to stereotype-congruent (*M* = 807 ms) than stereotype-incongruent pairings (*M* = 920 ms), *t*_1_ (35) = 3.76, *p* = .001, *dz* = .63; *t*_2_ (23) = 5.14, *p* < .001, *dz* = 1.05, while RTs to neutral pairings (*M* = 846 ms) fell between these two points. A marginally significant interaction of Congruency by Block was found in the by-participants analysis, *F*_1_ (3.47, 117.91) = 2.31, *p* = .07, while this interaction was highly significant in the by-items analysis; *F*_2_ (6, 99) = 3.20, *p* = .007, η_p_^2^ = .16. The pattern of RTs across blocks in each of the three congruency conditions (for the by-participants analysis) is shown in Figure 4.

#### Generalisability

To investigate whether the feedback training facilitated responding to a novel set of stereotype-incongruent role nouns, pre-training RTs (Block 1) versus post-training RTs (Block 3) were examined. RTs to stereotype-incongruent pairings significantly decreased by 327 ms across the study from Block 1 to Block 3, *t*_1_ (35) = 4.78, *p* < .001, *dz* *=* .94; *t*_2_ (23) = 7.33, *p* < .001, *dz* *=* .93, suggesting that performance-related feedback does facilitate responding to a novel set of stereotyped items in this judgement task. However, as explained in Experiment 1, practice effects may also have contributed to this effect. Indeed, this seems likely as RTs to stereotype-congruent pairings also improved significantly from Block 1 to Block 3, *t*_1_ (35) = 5.58, *p* < .001, *dz* *=* .93; *t*_2_ (23) = 7.12, *p* < .001, *dz* *=* 1.45. Ultimately, however, it was found that the congruent/incongruent difference in RTs was reduced from Block 1 to Block 3, *t*_1_ (35) = 2.22, *p* = .033, *dz* *=* .37; *t*_2_ (23) = 2.42, *p* = .024, *dz* *=* .49.

#### Durability

To investigate the durability of the training effect, RT performance between stereotype-incongruent word pairs in Block 3 and Block 4 was compared. No significant difference between these two blocks was found, *t*_1_ (35) = 0.31, *p* = .758; *t*_2_ (23) = 0.64, *p* = .526, with RTs at a very similar level in both (813 vs 804 ms, respectively). However, as the Block 4 RTs did not deteriorate across the week, this is again convincing evidence for the durability of the feedback training, with RTs still significantly lower than the pre-training levels of Block 1. Also, as with Block 1 to Block 3 above, a significant reduction in the congruent/incongruent difference was again found from Block 1 to Block 4, *t*_1_ (35) = 5.06, *p* < .001, *dz* *=* .84; *t*_2_ (23) = 2,35 *p* = .028, *dz* *=* .48. To confirm the durability of the training effect, we calculated Bayes factors comparing complete durability (no change in incongruent pair RTs from Block 3 to Block 4) to no durability (incongruent pair RTs reverting to Block 1 levels, 327 ms slower than in Block 3). Anything less than reversion to the original time disadvantage for incongruent items indicates some durability. The Bayes factors (BF0) were .12 by participants and .14 by items, providing evidence for no change from Block 3 to Block 4.

#### Fillers

Details of the analyses of the fillers can be found in the online Supplementary Material 2. As with Experiment 1, participants showed higher levels of accuracy to definitionally matching word pairs (*M* = 95.69%) than definitionally mismatching word pairs (*M* = 91.06%), with the lower mismatch scores again more apparent in response to male role terms (*M* = 86.09%) than female role terms (*M* = 96.04%). Similarly, participants were faster to respond to definitionally matching word pairs (*M* = 909 ms) than definitionally mismatching word pairs (*M* = 1,004 ms), with the slower mismatch times found in response to the male mismatch pairs (*M* = 1,048 ms) than the female mismatch pairings (*M* = 961 ms). As before, it is posited that this relatively poor performance to male mismatch fillers is explained by participants’ deliberation over certain role nouns that are male-specific by definition, but which are frequently used to refer to both sexes. Again, once participants learned to become stricter in their interpretations of the role nouns, accuracy of male mismatch pairings improved 18.89% from Block 1 (73.06%) to Block 4 (91.94%) and were thus much closer to responding on female mismatch pairings where performance was high from the outset (Block 1 average of 93.61%). Similarly, male mismatch RTs decreased 349 ms from Block 1 (1,282 ms) to Block 4 (933 ms), with final RTs not quite as fast as responding to female mismatch pairings (average of 857 ms in Block 4), but much more in line with expectations.

### Discussion

Overall, Experiment 2 provides support for the use of performance-related feedback as a strategy for overcoming spontaneous gender activation when reading gender-biased role nouns in English, so as to reduce levels of stereotype application. It was found that both accuracy and RT scores improved significantly from Block 1 to Block 3, indicating that participants learned not only to exert control over stereotype biases on which they had previously received feedback (as was found in Experiment 1), but to further control spontaneous biases towards novel stereotyped role nouns. Furthermore, it was found that the high level of accuracy and fast RTs to stereotype-incongruent items in Block 3 were still evident 1 week later. These findings provide support for the use of performance-related feedback as a useful strategy for combating the spontaneous activation of stereotype bias in the longer term. Next, a combined analysis of Experiments 1 and 2 was carried out to further investigate performance across the studies.

## Experiments 1 and 2: combined analysis

In this section, the data from Experiments 1 and 2 were combined so as to ascertain (1) whether post-training performance was significantly better than control levels when new role nouns had been introduced and (2) whether the feedback training improved performance on a novel set of role nouns to the same extent as on those on which the training had been received. Data from Blocks 1–3 of each experiment were used to investigate these issues (Block 4 was unique to Experiment 2 and was consequently omitted from this analysis).

### Results

#### Analysis

The combined trimmed data from Experiments 1 and 2 for both accuracy of judgements and correct RTs were analysed using two mixed-design ANOVAs, as outlined for Experiment 1. However, in the by-participants analyses, Training condition was updated to include three levels (Feedback/Control/Novel) and was included as a between-participants, but within-items factor. The findings reported below focus on effects that involved the Training condition variable, and more specifically on performance on critical incongruent trials across experiments.

#### Accuracy

**Figure 5.**
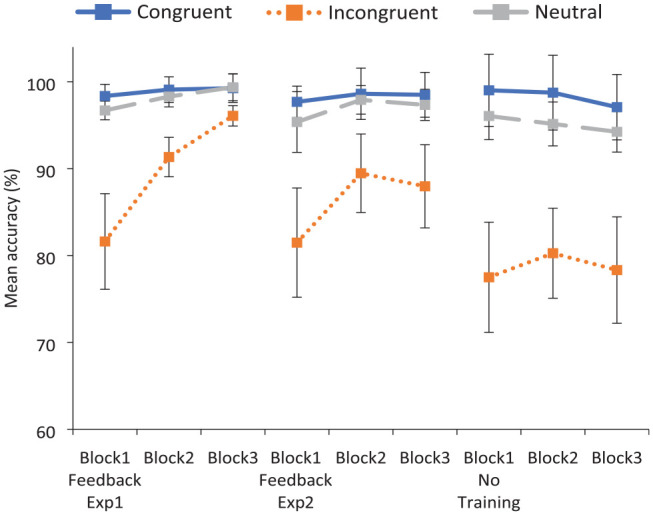
Mean accuracy to critical word pairs across blocks in all three conditions. Error bars indicate the 95% confidence intervals.

A main effect of Training condition was observed, *F*_1_(2, 111) = 3.25, *p* = .043, η_p_^2^ = .06; *F*_2_(2, 97) = 19.66, *p* *<* .001, η_p_^2^ = .29, with accuracy in the Feedback and Novel conditions (i.e., conditions in which training was provided) higher than in the Control (*M* = 95.1%, *M* = 93.8%, and *M* = 89.5%, respectively. Of more interest was a significant three-way interaction of Congruency by Block by Training condition, *F*_1_ (4.25, 235.88) = 3.23, *p* = .012, η_p_^2^ = .06; *F*_2_ (8, 194) = 4.60, *p* < .001, η_p_^2^ = .16. A graph of this interaction is shown in Figure 5. The Control condition is shown last, so as to best display the pattern of responding to incongruent word pairs across experimental conditions.

Unsurprisingly, the interaction of Congruency by Block by Training condition was primarily driven by variable performance on stereotype-incongruent trials, as ceiling effects were evident for stereotype-congruent and neutral pairings across conditions. To investigate performance on these stereotype-incongruent pairings in more detail, a second set of ANOVAs was conducted on the incongruent data alone to examine (1) Training condition (Control vs Novel) by Block (1 vs 3) performance, and (2) Training condition (Feedback vs Novel) by Block (1 vs 3) performance.

Despite similar levels of Block 1 accuracy in the Control vs Novel conditions, greater accuracy differences were found in Block 3: marginally different by-participants but significantly different by-items, *t*_1_ (54.47) = 1.44, *p* = .078; *t*_2_ (23) = 4.59, *p* < .001, *dz* = .94. This pattern of results again illustrates the superior accuracy performance after feedback training (this time to a novel set of role nouns) relative to the control condition in which no training was received.

Next a comparison of the Feedback versus Novel conditions revealed a significant Training condition by Block interaction (marginal by-participants), *F*_1_ (1, 85) = 3.53, *p* *=* .064; *F*_2_ (1, 23) = 11.79, *p* = .002, η_p_^2^ = .34. From Figure 5 it can be seen that Block 1 accuracy is almost identical in the two conditions (mean difference of .14%); however, one-tailed *t*-tests revealed that Block 3 accuracy was significantly different, *t*_1_ (40.35) = 2.00, *p* = .026, *d* = .63; *t*_2_ (23) = 5.00, *p* < .001, *dz* = 1.02, with the feedback training significantly more successful when the same role nouns were used post-training compared with a novel set.

#### RTs

**Figure 6.**
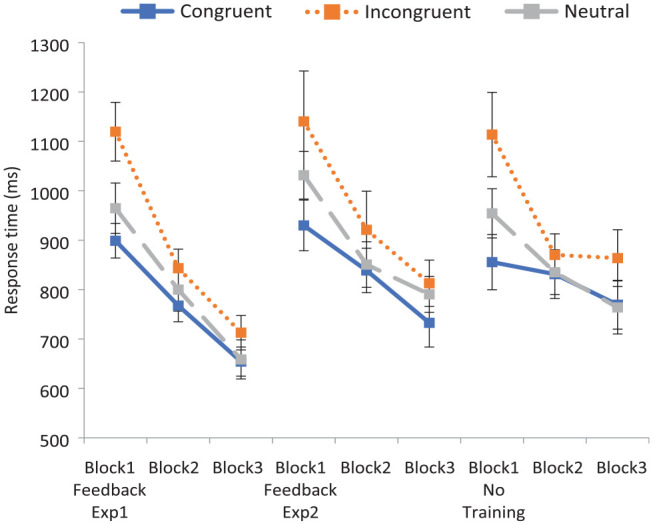
Mean response times to critical word pairs across blocks in all three conditions. Error bars indicate the 95% confidence intervals.

Contrary to expectations, no evidence of a Congruency by Block by Training condition interaction was found, *F*_1_ (8, 444) = .86, *p* *=* .55; *F*_2_ (8, 128) = .566, *p* = .804. However, to examine the pattern of responding across blocks, RTs to critical pairings in each of the three conditions are shown in Figure 6. The Control condition is again displayed last so as to best display the pattern of responding to incongruent word pairs across experiments.

Next, to more closely inspect the stereotype-incongruent data, a second set of ANOVAs was conducted on the incongruent data, investigating (1) Training condition (Control vs Novel) by Block (1 vs 3) performance, and finally (2) Training condition (Feedback vs Novel) by Block (1 vs 3) performance. However, there was no significant interaction of Training condition by Block interaction when considering the Control and Novel conditions, *F*_1_ (1, 64) = .72, *p* *=* .398; *F*_2_ (1, 23) = 1.70, *p* = .206. It appears that when novel items are introduced after the feedback training, final RTs are not significantly faster than in the control condition.

Finally, a comparison of the Feedback and Novel conditions also failed to reveal a significant Training condition by Block interaction, *F*_1_ (1, 85) = 1.28, *p* *=* .260; *F*_2_ (1, 23) = 1.67, *p* = .210. *T*-tests again found no difference between starting RTs in Block 1 (*p* > .7) across conditions; however, final Block 3 RTs were significantly different by-items, *t*_1_ (60.18) = 1.46, *p* = .149; *t*_2_ (23) = 2.50, *p* = .020, *dz* = .51. This last finding provides some evidence that the feedback training was more successful when the same role nouns were used post-training compared with a novel set, as was found with the accuracy data. However, it is worth noting that this pattern could be due to a pure repetition priming effect with participants improving more as they progressed through the task when they were receiving the same role nouns across blocks than different ones (as opposed to evidence that participants were necessarily learning to overcoming stereotypes more efficiently on the items on which they had previously received feedback).

### Discussion: combined analysis

A combined analysis of Experiments 1 and 2 was conducted to establish whether (1) post-training performance was significantly better than control levels when new role nouns were introduced, and (2) whether the feedback training improved performance on a novel set of role nouns to the same extent as on those on which the training was received.

First, the pattern of responses accuracy was largely as predicted. Accuracy of stereotype-incongruent word pairs was significantly higher in the Feedback and Novel conditions (where feedback training was provided) relative to the Control. However, despite Experiment 2 suggesting that the feedback training significantly increased accuracy of responding to novel stimuli (relative to pre-feedback levels), the combined analysis showed that Block 3 accuracy was significantly higher when the same set of role nouns was presented after the feedback training as opposed to a new set. It can be concluded that performance-related feedback is a highly effective means of reducing occupational stereotype application when a participant is presented with specific items on which feedback was received. Stereotype application can also be lowered when novel items are introduced, although the effect is somewhat reduced.

In the RT data, RTs to novel incongruent pairings were not significantly faster than those in the control condition. However, final RTs in the Feedback condition were faster than those of the Novel condition. These findings are largely in line with those of the accuracy data and suggest that performance-related feedback is most effective as a specific stereotype-reduction strategy, and its effects generalise to novel stimuli with moderate success. Again, this greater improvement in the Feedback condition could be partly driven by repetition effects, with participants improving as they learn how to correctly respond to certain terms as opposed to necessarily overcoming the stereotype associations of these items.

## General discussion

The primary aim of these studies was to investigate the efficacy of performance-related feedback as a strategy for overcoming spontaneous stereotype activation, so as to reduce occupational stereotype application on a judgement task, and in particular to reduce the use of an occupation to infer a probable gender. In both experiments before feedback was provided, participants showed more difficulty (i.e., lower accuracy and slower RTs) when responding to stereotype-incongruent word pairs as opposed to stereotype-congruent or neutrally rated pairs. These findings support those of previous researchers who have suggested that occupational stereotype information is automatically elicited from single words (e.g., Banaji & Hardin, 1996; Oakhill et al., 2005), and that gender resolution can be difficult to achieve in language processing when gender-related expectancies from occupational stereotypes clash with explicitly stated gender information (e.g., Carreiras et al., 1996; Irmen, 2007; Kreiner et al., 2008). The results of Experiment 1 revealed that accuracy and RTs of judgements to stereotype-incongruent pairings improved significantly following feedback, thus confirming the use of performance feedback as an effective means of lowering stereotype use. Moreover, when novel items were introduced after the feedback training (Experiment 2), accuracy and RTs were still significantly better than before feedback was received, and these effects were also evident 1 week later. While these findings provide support for the generalisability and durability of the feedback training, a combined analysis across both experiments found that final accuracy and RTs to incongruent pairings were better when participants were responding to word pairs on which they had received feedback (Experiment 1) as opposed to novel items (Experiment 2).

Although the current studies provide further evidence for the malleability of occupational stereotype biases, they are also consistent with previous research that has documented the persistency of stereotyping effects (e.g., Dunning & Sherman, 1997; Finnegan et al., 2015a, 2015b; Oakhill et al., 2005; Reynolds et al., 2006). Despite improved performance to stereotype-incongruent pairings following the feedback training, the same level of effortlessly fast and accurate responding found with stereotype-congruent and neutral pairings was never achieved. Therefore, while results are promising in terms of reducing spontaneous occupational stereotyping for gender, scope for further improvement remains.

### Mechanisms behind stereotype change

In the RT data, there was evidence that participants responded faster over time independently of whether they had received feedback. It is therefore important to note a distinction between speeding up with practice and becoming less stereotyped with practice. While participants may naturally speed up over time due to the repetitive nature of a task at which they become more adept, becoming less stereotyped requires more deliberate and controlled processes. A person must initially expend effort on consciously dismissing stereotyped associations and may then gradually show less evidence of succumbing to such biases. It is posited that, with practice/repetition, a newer response to a particular stimulus can then come to dominate an old (automatic) response (Kawakami et al., 2000; Shiffrin & Schneider, 1977; Smith et al., 1988; Wyer & Hamilton, 1998).

In our control condition, where performance feedback was not provided, responding to stereotype-incongruent pairings suggested that practice helped only decision-making speed as there was no concurrent increase in accuracy, i.e., practice alone was not enough to trigger a reduction in stereotype application. Conversely, when feedback was received, there was a simultaneous increase in accuracy to incongruent pairings alongside the reduction in RTs. Practice in this case seemingly allowed participants to repeatedly try to control stereotyped responding. The crucial result is the difference in stereotypical responding to incongruent pairs during and after training compared with the control condition. The lack of change in the close-to-ceiling effects for the congruent pairs does not detract from this finding. Thus, the successful reduction of bias was not an effect of extended practice but, more specifically, of the opportunity for self-regulation control that was afforded by the practice once feedback training had been provided (see also Kawakami and colleagues (2000), for successful use of a strategy that involved substantial practice plus an induced self-regulation goal “to not stereotype”). However, such self-regulation cannot be fully disentangled from two further mechanisms that likely played important roles in overcoming stereotype biases in this study, namely awareness and motivation.

The provision of direct, performance-related feedback clearly alerted participants to incorrect responding, and hence produced an awareness of biased responding. Wilson and Brekke (1994) posit that, to overcome mental contamination (i.e., unwanted judgements or behaviour induced by mental processing that is unconscious or uncontrollable), an awareness of this unwanted processing is first required. Then, in conjunction with the motivation and capacity to adjust responding, and knowledge of the direction and magnitude of adjustment required, participants should be able to successfully correct for the impact of the initial unwanted processing. The fact that participants were made explicitly aware of stereotyped responding in the current task is thus likely to have been the first critical step towards appropriately adjusting behaviour to overcome any activated bias.

Although the roles of motivation and awareness are confounded in this study, so we cannot dissociate the relative impact of these elements, our findings support past claims that once participants are alerted to a possible influence on their judgements and behaviour, and are motivated to overcome this influence, then they can do so to a large extent (e.g., Devine, 1989; Pratto & Bargh, 1991). Moreover, the opportunity for practice allowed participants to become adept at overcoming stereotyping. The provision of performance-related feedback is a neat strategy to engage the participant and allow for these mechanisms to operate in unison. Although our approach purposefully utilised multiple mechanisms, if we know what mechanisms work well together, future research can investigate how and why this is the case. This approach may prove more effective than examining all possible combinations of mechanisms with high levels of experimental control (Lai et al., 2014).

### Comparison with previous strategies

As mentioned briefly earlier, Lenton et al. (2009) conducted a meta-analysis on strategies used to overcome automatic stereotyping. They found that the type of intervention used was a significant moderator of success. Strategies based on (1) distracting or redirecting participants’ attention before category activation, or (2) facilitating the holding of multiple, different representations within the activated stereotype were typically more successful than those based on (3) stereotype prevention or inhibiting expression of stereotypes. While category (1) interventions were not suited to the present research, as we were interested in overcoming stereotype application *after* stereotype activation had already occurred through presentation of a gender-biased role noun, category (2) and (3) interventions were interlinked in our studies. The training strategy of performance feedback was devised to create awareness of category heterogeneity (category 2), while the judgement task itself involved inhibition or suppression of the stereotype bias so as to result in lower levels of stereotype use (category 3). Therefore, while the findings of this research do not provide conclusive evidence about the most successful type of intervention for stereotype reduction, they do provide support for the efficacy of highlighting category heterogeneity as a means of overcoming stereotype biases.

But how does performance in the current work compare with similar studies by Finnegan et al. (2015b) and Finnegan et al. (2015a), which both combined the judgement task used in this work with a strategy aimed at tackling spontaneous occupational stereotypes? To answer this question, we first compared effect sizes of the change in responding to stereotype incongruent trials from pre-training to post-training with data from Finnegan and colleagues (2015a, 2015b).^7^ Beginning with accuracy, we found that performance-related feedback led to slightly larger standardised effects (*dz* = 0.67—accuracy difference between Block 3 and Block 1 in Experiment 1) than presenting participants with counter-stereotype pictures (*dz* = 0.61) or providing them with social consensus-related feedback (*dz* = 0.35). A similar pattern was found in the by-items data with largest effects in the current study relative to the other two (*dz* = 2.24 vs *dz* = 1.16 and *dz* = 0.87, respectively). With the reaction time data, again, a larger effect size was found with performance feedback (*dz* = 1.47) than with the counter-stereotype pictures (*dz* = 0.77) or social consensus feedback strategies (*dz* = 0.61), which was mirrored in the by-items data (*dz* = 2.18 vs *dz* = 1.61 and *dz* = 1.88, respectively).^8^

The accuracy data is of most importance here. Improvement on the incongruent trials is found when training is provided, but absent when it is not. In the congruent condition, accuracy performance is close to ceiling across training conditions (97% or greater) so there is little room for improvement, but importantly, the gap between incongruent and congruent accuracy levels improves with training, but not with no training. In RT, there is a general speeding up across the experiment, regardless of the congruency or training condition. Practice effects appear to be at play here, unlike in the accuracy data. However, a smaller numerical difference is still found between the congruent and incongruent trials following training compared with when no training is provided. This pattern in the data suggests an effect of training in addition to practice effects.

Overall, the above comparisons with studies that employed similar designs and stimuli to the current work provide strong support for the value of performance-related feedback as a viable and effective means of stereotype reduction on the judgement task that is more effective than other strategies we have considered (Finnegan et al., 2015a, 2015b). Furthermore, this brief cross-study comparison highlights the importance of comparative evaluation of strategies aimed at stereotype reduction to determine which approaches are stronger than others, as well as the inclusion of effect sizes to help gauge which approach may be the most effective. However, while such comparisons are useful for investigating whether past results were broadly replicated or differed in important ways from the current results, subtle differences in design and procedure between the original and current experiments limit the conclusions that can be drawn.

### Further considerations and future research

One possible limitation of the study is that participants in the Training condition of each experiment were told that they would receive performance-related feedback, and what this feedback would entail, before the study began. It is possible that this information alone may have prompted reduced stereotyping by increasing salience of the need to inhibit from the outset biases about which gender was likely to be found in which occupations. If true, then actual provision of the feedback would not be an essential component of the intervention, and improved performance should be immediately evident from Block 1 of the Training condition in comparison to a control condition. In Block 1 of both experiments, accuracy on the critical incongruent pairings was 81.6% (Experiment 1) and 81.4% (Experiment 2) for those who knew they would go on to receive the feedback training. Importantly, this level of performance was not significantly higher than for those who received no feedback in Experiment 1, where the Block 1 mean was 77.5% (Experiment 2 had no control condition).

Similar results were found by Oakhill et al. (2005), Experiment 2, which was closely related to our No Training control condition and differed in only very minor ways (they included extra filler items, did not break their trials into separate blocks, and they presented the initial role noun on-screen for a shorter period of time (500 vs 1,000 ms in the current studies). In their study, performance on incongruent pairings was 79.2%, i.e., a difference of just 2.4% and 2.2% between their study and our Experiments 1 and 2, respectively, in the conditions where this feedback was provided. As noted above, while it is not ideal to make cross-experiment comparisons, together, these findings suggest that initial performance to critical stereotype-incongruent pairings was not meaningfully improved in Block 1 of the current work by alerting participants to the fact that they would receive feedback (and thereby potentially increasing the salience of needing to control gender-biased responding). We thus contend that that information alone was not sufficient to instigate stereotype change and that it is the feedback manipulation that led to the reduced stereotype application.

A concern highlighted about past research is the over-reliance on single-session laboratory-based studies in the stereotype reduction literature. Such research is problematic, because it does not allow a distinction between a temporary change in behaviour (malleability) and a longer-lasting change in underlying representations. An attempt was made to address this issue in Experiment 2, in which stereotype reduction was examined 1 week after the initial training. However, while the reduced levels of stereotyping suggest that there was a real change in the underlying stereotypes, it cannot be unequivocally confirmed that this was the case. Participants may simply have learned how to respond correctly in this highly specific experimental context, yet they may still succumb to occupational stereotype biases in different contexts, for example, when evaluating CVs or hiring people for jobs. Future research could therefore seek to combine the feedback training with a later session that assessed stereotype application in a different context, using a different task. The link between both sessions could be disguised to ensure participants were not overtly alerted to the fact that they should avoid occupational stereotyping in the second session. Such an approach would allow for a thorough evaluation of the feedback strategy in reducing stereotype use.

Clearly, examining and understanding the time course of an intervention’s efficacy can also have important implications for its application. While strategies that induce temporary change could prove useful for immediate use in specific social contexts, those that induce long-term change may help reduce discrimination in a broader range of contexts (Lai et al., 2014). Therefore, although this assessment of the durability of our results was a promising step in the right direction, a more extensive investigation of durability is required. Indeed, the proven mutability of stereotyped and prejudiced associations suggests that this will be a fruitful path despite the practical challenges of longitudinal research.

Other possibilities for further research, less directly related to our current concerns, would be to investigate the application of two kinds of models mentioned in the Introduction to data from our task. Reinforcement learning models might provide insights into the time course of learning in studies of the kind we have carried out and perhaps give some indication of the returns for extra learning time for the kinds of methods we have used. Drift diffusion models are potentially less relevant because of the greater complexity of our task compared with tasks ideally suited to drift diffusion modelling (see, for example, Ratcliff & McKoon, 2008). Nevertheless, an investigation of whether such models can provide a good fit to our data, and of which parameters in those models were significant, could provide insights into stereotype activation in our task. More generally, even though standard drift diffusion models cannot be applied to our task, and therefore cannot answer the question in this case, such models indicate that there can be methods of ascertaining whether certain patterns of data reflect the effects of stereotype activation or those of various forms of response bias, and such methods could be important if definitive conclusions about stereotype activation are to be drawn.

Finally, a notable strength of performance feedback training is that the central tenet of the strategy can be easily implemented outside of a laboratory context. If people are corrected upon making erroneous judgements about who might be suitable to engage in a particular occupation, and thus alerted to their own stereotyping tendencies, such awareness should be a first step towards overcoming them. With accompanying motivation to tackle bias, and adequate self-regulation tendencies, success should follow. Thus, while it may not be practicable to provide people with trial-by-trial feedback on occupational stereotypes, feedback via more natural means may challenge entrenched representations and gradually work to weaken stereotyping. Calling attention to stereotyped thinking should help to gradually attenuate its frequency. However, with this approach consideration would need to be paid to the context in which stereotype-consistent thinking is challenged. It is not always appropriate to comment on another’s behaviour and such an approach would presumably be most effective when a person does not feel judged for their error. For instance, such biases could be helpfully explored in an educational setting or a business diversity-training context. The hope for the longer term would be that people, and perhaps more particularly children, would not think they were excluded from certain occupations for irrelevant reasons, such as gender.

Overall, the studies outlined in this article provide support for (1) the claims of previous researchers who posit that some combination of awareness and motivation is required to overcome immediate stereotype biases (e.g., Bargh, 1992, 1999; Hilton & von Hippel, 1996; Macrae & Bodenhausen, 2000), (2) the value of extensive practice in overcoming stereotyping, and (3) the importance of investigating beyond the immediate effects of a training strategy to explore the generalisability and durability of the reported findings. While most stereotype reduction studies investigate only the immediate effects of training (Lenton et al., 2009; Paluck & Green, 2009), the present studies demonstrated the value of verifying that training successfully extends to newly introduced stimuli and of assessing the durability of training effects. Only through such stringent testing of strategies will a truly useful means of stereotype reduction be identified and a shift away from research on “quick fix” methods can commence.

## Supplemental Material

sj-docx-1-qjp-10.1177_17470218231196861 – Supplemental material for Performance-related feedback as a strategy to overcome spontaneous occupational stereotypes Supplemental material, sj-docx-1-qjp-10.1177_17470218231196861 for Performance-related feedback as a strategy to overcome spontaneous occupational stereotypes by Eimear Finnegan, Alan Garnham and Jane Oakhill in Quarterly Journal of Experimental Psychology

## Notes

1. Our previous work suggests that congruency effects in accuracy and RT data are readily detected with 16 participants per study (Oakhill et al., 2005), and that changes in these effects with training or feedback can be detected with 30+ participants for each between-participants condition (Finnegan et al., 2015a, 2015b). In the training condition of Experiment 1, we included some extra participants as a precautionary measure, because the feedback was more formal and might be regarded as less engaging than in our previous studies.

2. We use the term *critical* to refer to stereotype biased and neutrally rated items and word pairs that include such an item.

3. We based the presentation sequence on our earlier studies (Finnegan et al., 2015a, 2015b; Oakhill et al., 2005). Experiment 3 of Oakhill et al. (2005) found match/mismatch effects with a Stimulus Onset Asynchrony (SOA) of 1,800 ms. As noted above, the response in our task, which requires integrating information from two words, is more complex than typical sequential priming tasks, where shorter SOAs produce stronger effects. In Kidder et al.’s (2018) meta-analysis of sequential priming, only 11 studies had an SOA of over 500 ms and those studies had other features that may have determined why the effects tended to be weaker. For this reason, any conclusion from that meta-analysis about the time over which effects can occur, even in simple sequential priming, can only be tentative.

4. No reliable main effects or interactions with this variable were found and so it is not considered further. Indeed, sex differences in performance were not anticipated in this study based on previous findings of Oakhill et al. (2005).

5. Note that these *t*-tests were two-tailed for Block 1 comparisons (as no difference in performance was hypothesised at this stage), yet one-tailed for Block 3 comparisons (as it was hypothesised that performance would be significantly poorer in the absence of training). This same procedure was followed with the RT data.

6. Given that there were many fewer participants than items in this experiment (81 participants vs 456 item pairs per participant), it is highly likely that this effect was only significant by-items because we had more power in the by-items analysis, and the standard errors of the condition means were, therefore, lower in the by-items analysis than in the by-participants analysis, given that the variances are roughly equal. A similar imbalance between subject numbers and item numbers occurs in Experiment 2 and consequently this kind of pattern (a significant effect by-items but not by-participants) regularly recurs. Significant effects by-items should also be easier to detect as the training condition varies within-items but between-subjects.

7. Both of these studies had an initial block of trials as in the current study, but Finnegan et al. (2015b) had just two blocks overall (as the training involved responding to counter-stereotypical pictures after Block 1 and did not directly involve the judgement task). Finnegan et al. (2015a) was more similar to the current study, i.e., it had three blocks of trials and provided feedback in Block 2. However, their feedback was based on social consensus with a participant’s response, as opposed to feedback based on performance accuracy. Therefore, participants in Finnegan et al. (2015b) would have had one block of trials less than in the other studies, meaning any effect of practice would be lessened for that work.

8. Note that Oakhill et al. (2005) also used the judgement task used in the current work with other strategies aimed at tackling spontaneous occupational stereotypes. However, differences in design and procedure between these experiments make comparisons more difficult. In the Oakhill et al. studies, the instructional manipulations are between experiments, with Experiments 1 and 2 providing baseline measures. The most obvious comparisons are with their Experiment 2, not Experiment 1, as that experiment, like Experiments 3–6, used sequential presentation of the two nouns, not simultaneous presentation. Because of the different timing parameters in the different experiments (including speeded responding in Experiments 5 and 6), comparisons of time to respond to stereotype-incongruent trials in the different experiments are uninformative in relation to the hypotheses of interest here.
