# Supplementary material for: Performance-related feedback as a strategy to overcome spontaneous occupational stereotypes
Source: Q J Exp Psychol (Hove). 2023 Sep 8;77(6):1312–31. doi: 10.1177/17470218231196861 (PMC11103928; doi:10.1177/17470218231196861)
Supplement: sj-docx-1-qjp-10.1177_17470218231196861 – Supplemental material for Performance-related feedback as a strategy to overcome spontaneous occupational stereotypes [file sj-docx-1-qjp-10.1177_17470218231196861.docx]

Supplementary Material for:

**Performance-related Feedback as a Strategy to Overcome Spontaneous Occupational Stereotypes**

Eimear Finnegan^1, 2^, Alan Garnham^1^, and Jane Oakhill^1^

^1^ School of Psychology, University of Sussex, Brighton, England

^2^ School of Psychological Sciences and Health, University of Strathclyde, Glasgow, Scotland

**Author note**

Eimear Finnegan is now at the School of Psychological Sciences and Health, University of Strathclyde, Glasgow, Scotland

Correspondence concerning this article should be addressed to Alan Garnham, School of Psychology, University of Sussex, Brighton, BN1 6GN. Email: [a.garnham@sussex.ac.uk](mailto:a.garnham@sussex.ac.uk). ORCID ID: [0000-0002-0058-403X](http://orcid.org/0000-0002-0058-403X)

**Supplementary Material 1A**

**Stereotype Role Nouns for Experiment 1**

Note that in Gabriel et al. (2008) ratings range from 1-100; the high ratings indicate masculinity, low ratings indicate femininity, and ratings of 50% indicate neutrality.

| **Male stereotyped role nouns** | | |  | **Female stereotyped role nouns:** | | |
| --- | --- | --- | --- | --- | --- | --- |
|  | Bias | *SD* |  |  | Bias | *SD* |
| Bricklayer | 88.24 | -11.26 |  | Beautician | 13.27 | -10.88 |
| President | 86.80 | -16.96 |  | Fortune teller | 18.82 | -12.91 |
| Boxer | 86.27 | -15.62 |  | Au pair | 19.39 | -16.38 |
| Mechanic | 85.20 | -10.35 |  | Secretary | 21.76 | -13.81 |
| Football coach | 84.71 | -15.15 |  | Dressmaker | 22.94 | -13.90 |
| Lorry driver | 82.75 | -15.50 |  | Cleaner | 25.29 | -13.32 |
| Hunter | 82.16 | -13.16 |  | Flight attendant | 27.20 | -13.56 |
| Factory manager | 79.41 | -14.06 |  | Social worker | 27.84 | -12.05 |
| Electrician | 79.22 | -17.98 |  | Model | 28.43 | -14.33 |
| Pilot | 77.84 | -13.76 |  | Nurse | 29.02 | -21.28 |
| Golfer | 77.45 | -12.62 |  | Chocolate lover | 29.22 | -15.47 |
| Politician | 77.14 | -14.43 |  | Birth attendant | 30.82 | -18.01 |

| **Neutral-rated role nouns** | | |
| --- | --- | --- |
|  | Bias | *SD* |
| Pedestrian | 49.80 | -03.74 |
| Proof reader | 50.39 | -15.74 |
| Author | 49.60 | -10.29 |
| Trainee | 49.41 | -08.81 |
| Neighbour | 50.78 | -15.47 |
| Gynaecologist | 49.22 | -19.68 |
| Jogger | 48.82 | -11.77 |
| Concert go-er | 48.43 | -09.67 |
| Relative | 52.04 | -08.41 |
| Office worker | 47.65 | -13.94 |
| Artist | 52.55 | -13.54 |
| Adolescent | 52.94 | -10.64 |

**Supplementary Material 1B**

**Filler Role Nouns for Experiments 1 and 2**

*Note.* Eight of the male and female definitional terms used in the matching condition were also repeated in the mismatching condition (those in bold font were not repeated)

| **Condition** | **Male Definitional Role nouns** | | **Female Definitional Role nouns** | |
| --- | --- | --- | --- | --- |
| **Matching** | Policeman | Steward | Landlady | Bride |
|  | Groom | Waiter | Heroine | Waitress |
|  | Postman | King | Mistress | Princess |
|  | Salesman | **Craftsman** | Spinster | **Mermaid** |
|  | Bachelor | **Prince** | Hostess | **Ballerina** |
|  |  |  |  |  |
| **Mismatching** | Policeman | Landlord | Landlady | Policewoman |
|  | Groom | God father | Heroine | Grandmother |
|  | Postman | Count | Mistress | Seamstress |
|  | Salesman | Gigolo | Spinster | Geisha |
|  | Bachelor | Baron | Hostess | Lesbian |
|  | Steward | Fireman | Bride | Matron |
|  | Waiter | Grandfather | Waitress | Baroness |
|  | King | Milkman | Princess | Nun |
|  | Son | Host | Stewardess | Step mother |
|  | Sir | Duke | Milkmaid | Maid of honour |
|  | Knight | Best man | Salesgirl | Barmaid |
|  | Master | Barman | Duchess | Wife |
|  | Pope | Step brother | Countess | Queen |
|  | Hero | Step father | Dame | Madam |
|  | Husband | Priest | God mother | Daughter |

**Supplementary Material 1C**

**Stereotype Role Nouns for Experiment 2: Sets of items were individually paired based on strength of bias.**

The items previously used in Experiment 1 are numbered 1-6, while novel items for Experiment 2 are numbered 7-12 in each set. Items taken from Kennison and Trofe (2003) are presented in bold font; all others were sourced from Gabriel et al. (2008). In the former case, average ratings of both male and female participants are provided along with the standard deviations. The ratings of female participants are shown on the left-hand side while those of the male participants are shown on the right-hand side. In Gabriel et al. (2008) ratings range from 1-100; the high ratings indicate masculinity, low ratings indicate femininity, and ratings of 50% indicate neutrality. In Kennison and Trofe (2003) ratings range from 1-7; high ratings indicate masculinity and low ratings indicate femininity (neutral terms were chosen as close to 3.5 as possible)

**Male stereotyped role nouns:**

|  |  | **Set 1** | |  |  |  | **Set 2** | |
| --- | --- | --- | --- | --- | --- | --- | --- | --- |
| 1 |  | **Bias** | ***SD*** |  |  |  | **Bias** | ***SD*** |
| 1 | Bricklayer | 88.24 | -11.26 |  | 1 | President | 86.80 | -16.96 |
| 2 | Mechanic | 85.20 | -10.35 |  | 2 | Boxer | 86.27 | -15.62 |
| 3 | Football coach | 84.71 | -15.15 |  | 3 | Lorry driver | 82.75 | -15.50 |
| 4 | Factory manager | 79.41 | -14.06 |  | 4 | Hunter | 82.16 | -13.16 |
| 5 | Electrician | 79.22 | -17.98 |  | 5 | Pilot | 77.84 | -13.76 |
| 6 | Politician | 77.14 | -14.43 |  | 6 | Golfer | 77.45 | -12.62 |
| 7 | Engineer | 77.00 | -13.13 |  | 7 | Farmer | 76.80 | -14.49 |
| 8 | Mathematician | 74.90 | -12.06 |  | 8 | Carpenter | 75.49 | -18.15 |
| 9 | Murderer | 74.40 | -13.12 |  | 9 | Physicist | 73.53 | -12.62 |
| 10 | Judge | 72.75 | -14.43 |  | 10 | Butcher | 73.33 | -22.15 |
| 11 | Technician | 72.75 | -15.63 |  | 11 | Inventor | 71.63 | -12.31 |
| 12 | Prisoner | 71.57 | -12.06 |  | 12 | Statistician | 71.60 | -13.76 |

**Female stereotyped role nouns:**

|  |  | **Set 1** | |  |  |  | **Set 2** | |
| --- | --- | --- | --- | --- | --- | --- | --- | --- |
|  |  | **Bias** | ***SD*** |  |  |  | **Bias** | ***SD*** |
| 1 | Beautician | 13.27 | -10.88 |  | 1 | Fortune teller | 18.82 | -12.91 |
| 2 | Secretary | 21.76 | -13.81 |  | 2 | Au pair | 19.39 | -16.38 |
| 3 | Dressmaker | 22.94 | -13.90 |  | 3 | Cleaner | 25.29 | -13.32 |
| 4 | Social worker | 27.84 | -12.05 |  | 4 | Flight attendant | 27.20 | -13.56 |
| 5 | Model | 28.43 | -14.33 |  | 5 | Nurse | 29.02 | -21.28 |
| 6 | Birth attendant | 30.82 | -18.01 |  | 6 | Chocolate lover | 29.22 | -15.47 |
| 7 | Sales Assistant | 32.55 | -13.54 |  | 7 | Dancer | 33.53 | -14.54 |
| 8 | Hairdresser | 34.60 | -21.59 |  | 8 | Cashier | 33.67 | -15.23 |
| 9 | Dietician | 34.71 | -15.54 |  | 9 | Ice Skater | 34.80 | -12.66 |
| 10 | Violinist | 42.35 | -13.80 |  | 10 | Librarian | 35.00 | -16.07 |
| 11 | Singer | 44.80 | -11.47 |  | 11 | Cook | 45.10 | -18.91 |
| 12 | Secretary | 21.76 | -13.81 |  | 12 | **Florist** | **1.95 / 2.15** | **0.76 / 1.14** |

**Neutral-rated role nouns:**

|  |  | **Set 1** | |  |  |  | **Set 2** | |
| --- | --- | --- | --- | --- | --- | --- | --- | --- |
|  |  | **Bias** | ***SD*** |  |  |  | **Bias** | ***SD*** |
| 1 | Pedestrian | 49.80 | -03.74 |  | 1 | Proof reader | 50.39 | -15.74 |
| 2 | Trainee | 49.41 | -08.81 |  | 2 | Author | 49.60 | -10.29 |
| 3 | Neighbour | 50.78 | -15.47 |  | 3 | Gynaecologist | 49.22 | -19.68 |
| 4 | Concert go-er | 48.43 | -09.67 |  | 4 | Jogger | 48.82 | -11.77 |
| 5 | Relative | 52.04 | -08.41 |  | 5 | Office worker | 47.65 | -13.94 |
| 6 | Cinema go-er | 46.60 | -08.23 |  | 6 | Artist | 52.55 | -13.54 |
| 7 | Swimmer | 53.14 | -09.48 |  | 7 | Musician | 53.92 | -12.34 |
| 8 | Skier | 55.88 | -09.42 |  | 8 | Bank Clerk | 54.60 | -14.60 |
| 9 | Spectator | 54.71 | -12.86 |  | 9 | **Tour guide** | **4.00 / 3.95** | **0.79 / 1.00** |
| 10 | **Entertainer** | **4.05 / 4.15** | **0.22 / 0.49** |  | 10 | **Customer** | **3.75 /4.05** | **0.72 / 0.51** |
| 11 | **Patient** | **4.00 / 4.15** | **0.56 / 0.88** |  | 11 | **Photographer** | **4.00 / 3.80** | **0.79 / 0.52** |
| 12 | **Journalist** | **4.15 / 3.90** | **1.27 / 1.21** |  | 12 | **Acrobat** | **3.85 / 4.15** | **1.42 / 1.42** |

**Supplementary Material 2**

**Analysis of Filler Trials**

See the main text for a descriptive account of the points of interest from the findings reported below.

Experiment 1 – Accuracy for Filler Trials

Table S1: Mean Accuracy for the filler trials in Experiment 1 (by-participants SEM in parentheses)

Training Control

Stereo MM Match Mismatch Match Mismatch

Block1 Male Kinship .975(.008) .737(.017) .957(.011) .731(.022)

Female Kinship .947(.014) .929(.009) .943(.019) .934(.012)

Block2 Male Kinship .984(.009) .837(.020) .947(.011) .744(.026)

Female Kinship .963(.011) .938(.011) .940(.014) .924(.015)

Block3 Male Kinship .965(.011) .905((.019) .940(.015) .760(.025)

Female Kinship .945(.011) .953(.010) .953(.014) .937(.013)

.

There were significant effects of Congruity (*F*1 (1, 79) = 73.53, *p* < .001, *η_p_^2^* = 48; *F*2 (1, 76) = 8.23, *p* < .01. *η_p_^2^* = .10), Gender of Kinship Term (*F*1 (1, 79) = 97.47, *p* < .001, *η_p_^2^* = 55; *F*2 (1, 76) = 4.41, *p* < .05, *η_p_^2^* = .06), Training Condition (*F*1 (1, 79) = 8.17, *p* < .01, *η_p_^2^* = 09; *F*2 (1, 76) = 16.23, *p* < .001. *η_p_^2^* = .18), Training Condition by Kinship Term (*F*1 (1, 79) = 11.20, *p* = .001, *η_p_^2^* = 12; *F*2 (1, 76) = 9.51, *p* < .01. *η_p_^2^* = .11), Block (*F*1 (1.91, 150.86) = 12.51, *p* < .001, *η_p_^2^* = 14; *F*2 (1.65, 125.03) = 5.08, *p* < .05. *η_p_^2^* = .06), Block by Congruity (*F*1 (1.87, 147.79) = 13.36, *p* < .001, *η_p_^2^* = 15; *F*2 (1.65, 125.03) = 7.04, *p* < .01. *η_p_^2^* = .09), Block by Kinship Term by Congruity (*F*1 (1.80, 141.96) = 14.46, *p* < .001, *η_p_^2^* = .16; *F*2 (1.65, 125.03) = 5.18, *p* < .05. *η_p_^2^* = .06), Training Condition x Block (*F*1 (1.91, 150.86) = 8.89, *p* < .001, *η_p_^2^* = 10; *F*2 (1.63, 124.15) = 6.21, *p* < .01. *η_p_^2^* = .08), Training Condition by Block by Kinship Term (*F*1 (2, 158) = 6.66, *p* < .01, *η_p_^2^* = 08; *F*2 (1.63, 124.15) = 3.85, *p* < .05. *η_p_^2^* = .05), and Training Condition by Block x Congruity (*F*1 (1.87, 147.79) = 6.31, *p* < .01, *η_p_^2^* = .07; *F*2 (1.63, 124.15) = 5.72, *p* < .01. *η_p_^2^* = .07).

Experiment 1 – Response Times for Filler Trials

Table S2: Mean Response Time (milliseconds) for the filler trials in Experiment 1 (by-participants SEMs in parentheses)

Training Control

Stereo MM Match Mismatch Match Mismatch

Block1 Male Kinship 1072(41.12) 1175(38.67) 959(54.09) 1163(50.87)

Female Kinship 1064(40.28) 1089(37.61) 991(52.98) 1076(49.47)

Block2 Male Kinship 914(36.87) 961(34.13) 913(48.49) 1023(44.89)

Female Kinship 899(34.62) 920(33.58) 903(45.54) 947(44.17)

Block3 Male Kinship 866(34.82) 871(31.42) 893(45.81) 935(41.33)

Female Kinship 832(31.52) 827(26.88) 857(41.46) 856(35.36)

.

There were significant effects of Congruity (*F*1 (1, 77) = 14.93, *p* < .001, *η_p_^2^* = .16; *F*2 (1, 76) = 10.63, *p* < .01. *η_p_^2^* = .12), Gender of Kinship Term (*F*1 (1, 77) = 11.08, *p* < .001, *η_p_^2^* = .13; *F*2 (1, 76) = 5.89, *p* < .05. *η_p_^2^* = .07), Block (*F*1 (1.88, 144.50) = 60.09, *p* < .001, *η_p_^2^* = .44; *F*2 (1.98, 150.10) = 132.41, *p* < .001. *η_p_^2^* = .64), Congruity x Block (*F*1 (2, 154) = 6.52, *p* < .01, *η_p_^2^* = .08; *F*2 (1.98, 150.10) = 8.42, *p* < .001. *η_p_^2^* = .10), Block by Training Condition, marginal by-P significant by-I (*F*1 (1.88, 144.50) = 3.08, *p* = .052, *η_p_^2^* = .04; *F*2 (1.80, 137.04) = 3.84, *p* < .05. *η_p_^2^* = .05), Congruity by Kinship Term by-P only (*F*1 (1, 77) = 8.38, *p* < .01, *η_p_^2^* = .10; *F*2 (1, 76) = 2.20, n.s)*.,* and Congruity by Training Condition by-I only (*F*1 (1, 77) = 2.68, n.s; *F*2 (1, 76) = 9.42, *p* < .01. *η_p_^2^* = .11).

Experiment 2 – Accuracy for Filler Trials

Table S3: Mean Accuracy for the filler trials in Experiment 2 (by-participants SEMs in parentheses)

Male Kinship Female Kinship

Match Mismatch Match Mismatch

Block1 . .967(.014) .731(.024) .919(.024) .936(.013)

Block2 . .969(.012) .875(.013) .958(.018) .964(.007)

Block3 .964(.011) .919((.016) .964(.014) .965(.007)

Block 4 .967(.010) .919(.014) .947(.016) .977(.005)

.

There were significant effects of Gender of Kinship Term (*F*1 (1, 34) = 29.61, *p* < .001, *η_p_^2^* = .47; *F*2 (1, 76) = 4.41, *p* < .05. *η_p_^2^* = .06), Congruity (*F*1 (1, 34) = 14.12, *p* < .001, *η_p_^2^* = .29; *F*2 (1, 76) = 8.23, *p* < .01. *η_p_^2^* = .10), Gender by Congruity (*F*1 (1, 34) = 122.48, *p* < .001, *η_p_^2^* = .78; *F*2 (1, 76) = 6.17, *p* < .05. *η_p_^2^* = .08), Block (*F*1 (2.861, 97.27) = 27.61, *p* < .001, *η_p_^2^* = .45; *F*2 (3, 228) = 10.83, *p* < .001. *η_p_^2^* = .125), Block by Congruity (*F*1 (2.49, 84.60) = 7.35, *p* < .001, *η_p_^2^* = .18; *F*2 (3, 228) = 5.71, *p* < .001. *η_p_^2^* = .07), and Block by Kinship Term by Congruity (*F*1 (2.96, 100.73) = 20.57, *p* < .001, *η_p_^2^* = .38; *F*2 (3, 228) = 6.01, *p* < .05. *η_p_^2^* = .07. The interaction of Gender by Block was significant by participants, but not by items (*F*1 (2.74, 93.08) = 5.69, *p* < .01, *η_p_^2^* = .14; *F*2 (3, 228) = 2.15, *n.s*., *η_p_^2^* = .03)

Experiment 2 – Response Times for Filler Trials

Table S4: Mean Response Time (milliseconds) for the filler trials in Experiment 2 (by-participants SEMs in parentheses)

Male Kinship Female Kinship

Match Mismatch Match Mismatch

Block1 . 1013(44.55) 1253(63.43) 974(43.04) 1134(54.72)

Block2 . 909(50.43) 1048(59.75) 919(57.31) 975(54.02)

Block3 982(54.56) 942((42.52) 848(46.95) 894(40.82)

Block 4 863(50.91) 940(45.66) 812(40.21) 858(40.73)

.

There were significant effects of Congruity (*F*1 (1, 35) = 15.25, *p* < .001, *η_p_^2^* = .30; *F*2 (1, 76) = 17.22, *p* < .001. *η_p_^2^* = .19), Gender of Kinship Term (*F*1 (1, 35) = 24.37, *p* < .001, *η_p_^2^* = 41; *F*2 (1, 76) = 9.68, *p* < .01. *η_p_^2^* = .11), Block (*F*1 (2.69, 94.14) = 19.51, *p* < .001, *η_p_^2^* = .36; *F*2 (3, 228) = 68.08, *p* < .001. *η_p_^2^* = .47), Block by Congruity (*F*1 (2.44, 85.22) = 7.74, *p* < .001, *η_p_^2^* = .18; *F*2 (3, 228) = 13.20, *p* < .001. *η_p_^2^* = .15), and Block by Kinship Term by Congruity by-I only (*F*1 (2.94, 103.00) = 2.61, n.s; *F*2 (3, 228) = 3.53, *p* < .05. *η_p_^2^* = .04).
